# How health systems contribute to societal goals

**DOI:** 10.2471/BLT.24.291809

**Published:** 2024-06-04

**Authors:** Rachel Greenley, Dheepa Rajan, Kira Koch, Josep Figueras

**Affiliations:** a London School of Hygiene and Tropical Medicine, Keppel Street, London WC1E 7HT, England.; b European Observatory on Health Systems and Policies, Brussels, Belgium.; c Special programme on Primary Health Care, World Health Organization, Geneva, Switzerland.

Traditionally, health system performance assessments have focused on evaluating the health system itself, with less concern about the broader impacts of improved population health beyond the sector. However, in today’s interconnected world, health is intricately linked with the environment, sociocultural dynamics, geopolitics and the economy, among others. These interconnections highlight the need for health system performance assessments to recognize that achieving health goals can also contribute to broader societal objectives, including population well-being, economic development, environmental sustainability and social cohesion.

Recent health policy discussions have explored, for example, the environmental footprint of health systems, the effects of social factors such as loneliness on well-being, the breakdown of trust in politicians during events such as the coronavirus disease 2019 (COVID-19) pandemic, and the economic implications of mental health on employment and poverty status. These discussions underline that health systems are responsible for providing health services and have a pivotal role in the improvement of people’s health, promotion of overall well-being, happiness and productivity. 

To better understand the contribution of health systems to broader societal well-being, such goals need to be adequately conceptualized and measured. Well-being is not captured by standard economic measures such as gross domestic product (GDP), which primarily focus on economic growth and fail to reflect income distribution, sustainability practices, non-market transactions and health and education outcomes, many of which contribute to societal well-being. Newer, more holistic measurement approaches to well-being, for example the Organisation for Economic Co-operation and Development’s (OECD) well-being framework,^1^ attempt to quantify well-being through various factors such as health, education, employment, housing, security, gender equality and social connections.

In this article, we conceptualize societal well-being from the perspective of the health system’s contribution to it. We break down well-being into three societal goals – social cohesion, environmental sustainability and economic development (Fig. 1). The health system contributes to these objectives through actions that primarily serve to achieve its own goals such as improving population health, equity, people-centredness or resilience. Therefore, achieving health system goals leads to considerable contributions to societal goals.

**Fig. 1:**
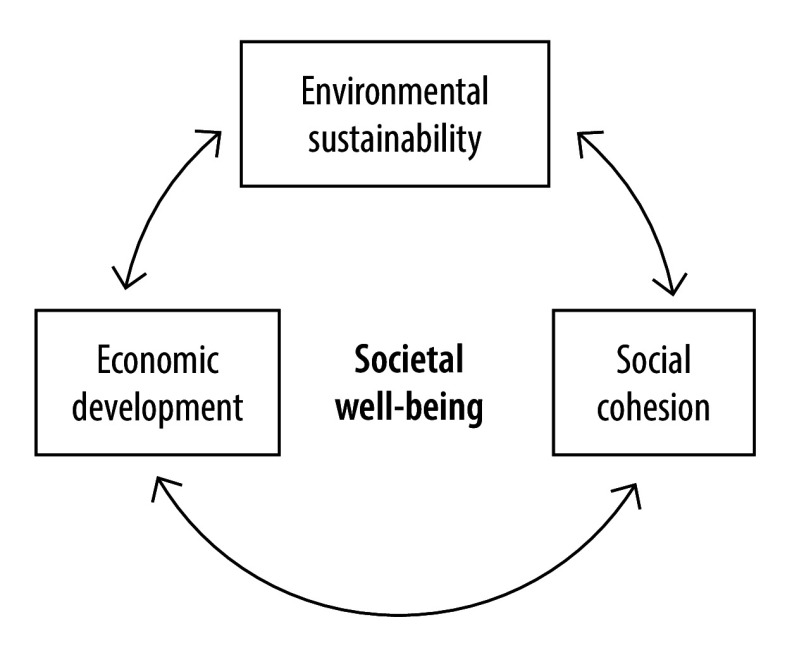
The goals of societal well-being

## Conceptualizing societal well-being

Societal well-being represents an aggregate measure of societal (or the population’s) overall quality of and satisfaction in life, encapsulating emotional, environmental, social and economic dimensions. Health systems contribute value to people’s lives and thus contribute to well-being – for example through curative care but also through preventive and promotive services, through public health functions and through various other actions it undertakes, such as community engagement.

Those services and actions aim to ensure good health, including mental health, which has been consistently identified as an important contributor to both individual and population (societal) well-being.^1^

### Social cohesion

Social cohesion is the bond that holds a society together, manifesting in trust, solidarity and a collective commitment to shared values and objectives.^2^^,^^3^ The health system fosters social cohesion by ensuring equitable access to health care, involving communities in health initiatives, reducing health disparities and promoting inclusivity – key aspects of people-centred care. The health system goals of people-centredness and equity promote trustworthiness.^3^ By building trust that the necessary care will be readily available for everyone, a sense of fairness and social responsibility is created in the community, leading to stronger social cohesion. For example, community health initiatives that involve participatory approaches for health promotion and disease prevention can enhance community ties and mutual support, leading to better health outcomes and lower mortality rates over time.^4^ Social cohesion also contributes to local community development, which often depends on a community’s ability to agree on common goods to be created for the benefit of its community members.^2^ More specifically, inclusive community health initiatives involve members of vulnerable and marginalized groups in the planning and implementation of health strategies with aims for eliminating discrimination, reducing income inequality, reducing barriers and ensuring equal access to health care.

### Economic development

This societal goal encompasses processes that enhance living standards, create jobs and spur innovation – elements that standard economic metrics fail to capture. This dimension is vital for providing necessary resources for health and improving overall quality of life. The health system contributes to economic development through maintaining a healthy workforce, reducing absenteeism and burnout, promoting productivity and addressing health inequities to enable full economic participation. Notably, with a healthy workforce, the health sector’s proportion of GDP for OECD countries was reported at around 9.2% in 2022,^5^ representing a significant share of the economy. A health system goal of financial protection helps to protect individuals from catastrophic health expenditures through mechanisms such as prepayment and pooled resources, which protects them from falling into poverty due to health-care costs. This protection is linked to economic development as it helps to ensure productivity and economic contributions are maintained without the financial burden of health expenses. Consequently, a health system goal of health improvement (that is, a healthier workforce) boosts overall economic productivity and growth. The health sector’s growth has implications for the overall economic health of nations, especially given its size and expansion rate. Before the COVID-19 pandemic, the health sector was expanding more rapidly than the overall economy in OECD countries, causing the health share of GDP to increase nearly 1% in the years 2000–2018. This translated to current health spending sharply increasing, indicating a substantial increase in the financial resources dedicated to health care.^6^ Additionally, by addressing health inequities, marginalized and vulnerable members of the community can participate more fully in the economy. In essence, the health system acts as a recipient of economic resources and as an active player in shaping the economic landscape, fostering a resilient economy that underpins societal well-being.

### Environmental sustainability

Environmental sustainability describes responsible interactions with the planet to preserve its resources. Climate change is expected to considerably influence health system usage and the need for service transformation, as the health sector’s negative impact on the ecological footprint is now recognized. In 2019, a study estimated that, if health care were a country, it would be the fifth largest emitter of carbon emissions worldwide.^7^ As a result, health system contributions to environmental sustainability have begun through the optimization of health service resources, as well as greening initiatives that reduce its carbon footprint and other environmental impacts. Additionally, the health system has adapted services and structures to better respond to emerging climate-related issues such as an increased number of catastrophic events or vector-borne diseases.^8^ The health system goal of efficiency is a means of contributing to environmental sustainability through ongoing efforts to optimize resource use, reduce waste and adapt to the changing needs of a population in climate crises, to provide care more efficiently. The sustainable management of our environment is thus not an isolated endeavour but is intrinsically linked to our societal well-being, economic development and the pursuit of social cohesion. 

### Intersections

Every goal within society, while significant on its own in the context of health system performance, does not encompass or support alone the complex needs of society. For instance, the attainment of social cohesion hinges upon a foundation of trust and solidarity, prompting a community to embrace behaviours that promote health and solidarity,^9^ which in turn lay the groundwork for economic growth. Rather, social cohesion acts as a precursor for economic development.^10^ Social solidarity plays an important role in the beliefs and attitudes around climate mitigation strategies or conservation efforts.^11^ Environmental sustainability requires collective, community action. The intricate relationship between social and environmental factors underscores the need for a holistic approach. Achieving equity necessitates active participation, a core principle of environmental justice movements.^12^ Engaging people, communities and civil society in decision-making fosters fairness and strengthens social cohesion and sustainability.^13^ Equity comprises both distributive justice and procedural justice facets within a society, emphasizing the interconnectedness of social and environmental well-being. More broadly, these three goals relate to societal well-being as a proxy measure of quality of life within a resilient and healthy community.

In conclusion, health policy-makers evaluating their system’s performance should recognize that achieving certain health system goals can considerably enhance overall societal well-being. In this article, we make an initial effort to advance the discussion on this important topic. 

## References

[1] Boarini R, Kolev A, McGregor A. Measuring well-being and progress in countries at different stages of development. Paris: Organisation for Economic Co-operation and Development; 2014. 10.1787/5jxss4hv2d8n-en

[2] Schiefer D, Van der Noll J. The essentials of social cohesion: a literature review. Soc Indic Res. 2017;132(2):579–603. 10.1007/s11205-016-1314-5

[3] McKee M, Greenley R, van Schalkwyk M, Permanand G. Trust: the foundation of health systems. Copenhagen: WHO Regional Office for Europe on behalf of the European Observatory on Health Systems and Policies; 2024. [Forthcoming]. 40982613

[4] Kawachi I, Berkman LF. Social cohesion, social capital, and health. In: Berkman LF, Kawachi I, Glymour MM, editors. Social Epidemiology. 2nd ed. Oxford: Oxford University Press; 2014. 10.1093/med/9780195377903.003.0008

[5] Health at a glance 2023. Paris: Organisation for Economic Co-operation and Development; 2023. Available from: https://www.oecd.org/health/health-at-a-glance/ [cited 2024 Mar 21].

[6] Spending on health in Europe: entering a new era. Geneva: World Health Organization; 2021. Available from: https://www.who.int/europe/publications/i/item/9789289055079 [cited 2024 May 31].

[7] Karliner J, Slotterback S, Boyd R, Ashby B, Steele K, Wang J. Health care’s climate footprint: the health sector contribution and opportunities for action. Eur J Public Health. 2020;30 Supl5:ckaa165.843. 10.1093/eurpub/ckaa165.843

[8] WHO guidance for climate-resilient and environmentally sustainable health-care facilities. Geneva: World Health Organization; 2020. Available from: https://www.who.int/publications/i/item/9789240012226 [cited 2024 Mar 25].

[9] Han Q, Zheng B, Cristea M, Agostini M, Bélanger JJ, Gützkow B, et al. PsyCorona Collaboration. Trust in government regarding COVID-19 and its associations with preventive health behaviour and prosocial behaviour during the pandemic: a cross-sectional and longitudinal study. Psychol Med. 2023 Jan;53(1):149–59. 10.1017/S0033291721001306 33769242 PMC8144822

[10] Graeff P, Svendsen GT. Trust and corruption: the influence of positive and negative social capital on the economic development in the European Union. Qual Quant. 2013;47(5):2829–46. 10.1007/s11135-012-9693-4

[11] Bazzani G. Climate solidarity: a framework and research agenda for low–carbon behavior. Sociol Forum. 2023;38(2):352–74. 10.1111/socf.12885

[12] Schlosberg D. Defining environmental justice: theories, movements, and nature. Oxford: Oxford University Press; 2007. 10.1093/acprof:oso/9780199286294.001.0001

[13] Cuthill M. Strengthening the ‘social’ in sustainable development: Developing a conceptual framework for social sustainability in a rapid urban growth region in Australia. Sustain Dev. 2010;18(6):362–73. 10.1002/sd.397

